# Changes in physical performance according to job demands across three cohorts of older workers in the Longitudinal Aging Study Amsterdam

**DOI:** 10.1007/s10433-023-00768-9

**Published:** 2023-06-07

**Authors:** Mikaela B. von Bonsdorff, Matti Munukka, Natasja M. van Schoor, Monika E. von Bonsdorff, Lauri Kortelainen, Dorly J. H. Deeg, Sascha de Breij

**Affiliations:** 1 Faculty of Sport and Health Sciences and Gerontology Research Center, University of Jyväskylä, P.O. Box 35, 40014 Jyvaskyla, Finland; 2 Folkhälsan Research Center, Helsinki, Finland; 3 Faculty of Sport and Health Sciences, University of Jyväskylä, Jyvaskyla, Finland; 4 Epidemiology and Data Science, Amsterdam UMC, Location Vrije Universiteit Amsterdam, Amsterdam, The Netherlands; 5 Amsterdam Public Health, Aging and Later Life, Amsterdam, The Netherlands; 6 Jyväskylä Business School for Economics, University of Jyväskylä, Jyvaskyla, Finland; 7 Department of Health and Social Management, University of Eastern Finland, Kuopio, Finland

**Keywords:** Physical job demands, Psychosocial job demands, Physical performance, Cohort study, Ageing

## Abstract

This study set out to evaluate the association between job demands at baseline and physical performance over a six-year period across three cohorts of older Dutch workers examined 10 years apart. Data were drawn from three cohorts (1992–1999, 2002–2009 and 2012–2019) of the Longitudinal Aging Study Amsterdam. Individuals aged 55–65 years from each cohort who worked for pay were included (n = 274, n = 416, n = 618, respectively). Physical performance was measured using gait speed and chair stand performance. A population-based job exposure matrix was used to indicate levels of exposure probability of physical (use of force and repetitive movements) and psychosocial (cognitive demands and time pressure) job demands. We found that psychosocial job demands increased and physical demands decreased across the three cohorts. No between cohort differences were found for how job demands affected changes in physical performance over follow-up. For men, faster decline in gait speed was observed when comparing higher and lower use of force at baseline (β −0.012, 95% CI −0.021, −0.004). Greater use of force and repetitive movements were associated with faster decline in chair stand performance (β −0.012, 95% CI −0.020, −0.004 and β −0.009, 95% CI −0.017, −0.001, respectively). In women, no association of job demands on change in physical performance was observed. The study concluded that higher physical job demands were associated with stronger decline in physical performance across six years for men in all cohorts, while no associations were found among women.

**Supplementary Information:**

The online version contains supplementary material available at 10.1007/s10433-023-00768-9.

## Introduction

Population ageing increases the need for information of factors that influence the patterns of health and functioning in the older population (Christensen et al. 2009). Work is a major part of adulthood as both men and women spend a considerable proportion of their time at the workplace. Working conditions play an important role in health and functioning in older age (Wahrendorf et al. 2012; Nilsen et al. 2017). During the past decades, physical demands of work have decreased during the previous decades while psychological demands of work have increased, and work participation of older employees and women have increased (Eurofound 2006; Ng and Popkin 2012). Little is known about the potential influence of this shift of working conditions on health, functioning and performance and particularly on changes in these outcomes in recent cohorts of older workers.

Physical performance is an indicator of the ability of a person to function in everyday life and a good predictor of later health outcomes such as disability, increased need of healthcare services and premature mortality (Guralnik et al. 1995; von Bonsdorff et al. 2006; Cooper et al. 2010). Earlier studies have shown that change in physical performance across time is heterogeneous among older individuals (White et al. 2013; Hoekstra et al. 2020). This heterogeneity necessitates the assessment of a variety of factors including job exposures that may be related to change in physical performance across time.

Higher physical job demands, such as lifting, kneeling, and repetitive movements, have been linked with poor self-reported health and physical functioning in several cohort studies (Hinrichs et al. 2014; Platts et al. 2017; Møller et al. 2019). A few studies have reported on the association between physical job demands and objectively measured physical performance of which the findings have been inconsistent. A Danish cohort study showed a negative association between physical job demands and chair stand performance among middle-aged men but not women (Møller et al. 2015). A Swedish study showed that higher physical job demands were associated with poorer measures of isometric strength, physical fitness and dynamic endurance among women (Torgén et al. 1999). A Dutch study found no association between physical job demands and chair stand performance in analyses pooled by gender (van der Noordt et al. 2019).

Higher psychological job demands have been shown to be associated with poorer self-reported physical functioning in adulthood (Cheng et al. 2000; von Bonsdorff et al. 2014; Hansen et al. 2014; Stansfeld et al. 1998) and in older age (Wahrendorf et al. 2012). So far, only few studies have explicitly investigated psychosocial job demands and objectively measured physical performance in older employees showing no associations in analyses pooled by gender (van der Noordt et al. 2019) or stratified by gender (Nilsen et al. 2019).

The objective of this study was to evaluate whether associations between physical and psychological job demands at baseline and physical performance over a six-year period differed across three cohorts of older Dutch workers. We expect that the increase in work participation leads to a change in susceptibility to the effect of working conditions, as the ageing peers of people who exited from the workforce in earlier cohorts, in more recent cohorts continue working. In addition, the improvements in work conditions due to advances in occupational health and safety measures (Turek et al. 2020) may play a role in this development. For our study, we used unique data consisting of three measurement points in three cohorts that were assessed ten years apart.

## Methods

### Data

This study uses data from the population-based Longitudinal Aging Study Amsterdam (LASA), an ongoing study of changes in functioning of older adults aged 55 and above in the Netherlands, with follow-ups every three years. The sampling, data collection procedures and non-response have been described in detail earlier in the latest cohort profile update (Hoogendijk et al. 2020). Briefly, cohort 1 in 1992–1993 included 3107 older persons aged between 55 and 85 years, of which 966 were aged 55–65 years (response rate 62%). Cohort 2, started in 2002–2003, included 1002 (response rate 62%) and cohort 3, started in 2012–2013, included 1023 people aged 55–64 years (response rate 63%.

### Study sample

Individuals aged 55 to 65 in paid work ≥ 1 h per week at baseline were included (van der Noordt et al. 2019; Boot et al. 2014). This sample consisted of 1308 occupationally active respondents at baseline (cohort 1: n = 274; cohort 2: n = 416; cohort 3: n = 618). Due to item-non-response at baseline, the sample in this paper included 1093 participants (see Supplementary Table S1). The item-non-responders (n = 215) were not significantly different in terms of age, gender and educational level (all p-values > 0.247). We included three measurement waves for all cohorts. All together 333 participants dropped out at wave 2 or 3, but they did not differ according to age, gender, and educational level (all p-values > 0.18) from the ones who participated in all three waves (n = 975). 1093 participants were included in the study sample and drop-out was accounted for in the analyses. Follow-up consisted of 1497, 2324 and 3107 person-years for Cohorts 1, 2 and 3, respectively. The Medical Ethics Committee of the VU University Medical Center approved the LASA study; informed consent was obtained from all respondents.

### Measures

#### Physical performance

Two standardized tests on physical performance, timed measurements of gait speed and chair stand, were used as outcome measures across three time points for each cohort. Timed Chair Stand Test involved standing up without the use of arms five times at usual pace. Chair stand speed was defined as the number of chair rises per second. The chair stand test measures leg strength and has been shown to be a valid and reliable measure of functional mobility in a sample of older women (Goldberg et al. 2012). In the gait speed test, participants were asked to walk 3 m, turn around and walk back 3 m as quickly as possible without running. Time needed to complete the test was recorded to the nearest second using a stopwatch and the result was expressed as meters per second, with faster speed reflecting better performance. The gait speed test is a measure of functional status and overall health and has been found to be valid and reliable in diverse populations (Middleton et al. 2015).

#### Job demands

Job demands of the current job at baseline were derived from a general population job exposure matrix (GPJEM) for representative samples of 55- to 65-year-old workers (Rijs et al. 2014). This GPJEM indicates levels of exposure probability of physical and psychosocial job demands as the percentages low, intermediate, and high exposure within a job category. It has successfully been used in previous studies to determine work exposures and predict health effects (de Wind et al. 2020; Rijs et al. 2014). Physical job demands included the necessity to use force during work (i.e., use of a lot of force, such as in lifting, pushing, pulling or carrying or using force with work tools) and performing repetitive movements. Physical job demands were dichotomized into high (highest third) and low (two lowest thirds). Psychosocial job demands involved time pressure (i.e., working at high pace and working under high time pressure) and cognitive demands (e.g. intensive thinking, need to keep focused and requiring much concentration) (Rijs et al. 2014). Psychosocial job demands were dichotomized into high (highest third) and low (two lowest thirds) (Kulmala et al. 2014).

#### Covariates

We adjusted the analyses for age, socioeconomical status, body mass index and lifestyle factors as it has been shown in several studies that work demands and health behaviours each have independent, unique effects (Andersen et al. 2016; Lund and Csonka 2003; Schram et al. 2021). Sex and date of birth were obtained from municipal registries. Educational level was categorized into low (elementary education at most), middle (lower vocational and general intermediate education, Intermediate vocational education and general secondary education) and high (higher vocational education, college education and university). Body mass index (BMI) was calculated using the participant's measured height and weight. The number of alcohol consumption per week was categorized into none, moderate (men 1–3 and women 1–2 glasses/day) and high use (men at least 4 and women at least 3 glasses/day). Smoking status was categorized into never, former and current smoker. Physical activity was measured using the LASA Physical Activity Questionnaire, which covered frequency and duration of activities including walking outdoors, light household activities, heavy household activities and two most frequently performed sports performed in the past two weeks (Stel et al. 2004; Ainsworth et al. 2011) and defined as total metabolic equivalent of task (MET) based on hours/week spent on each activity. For work status, respondents were asked at each follow-up if they had a paid job at present, which included one or more hours of work per week. Thus, temporary unemployment or exit from the workforce during the follow-up period was accounted for.

### Statistical analyses

Descriptive analyses were performed to examine main characteristics of participants by cohort at baseline. For continuous variables mean and standard deviation were calculated for each cohort. Also, the differences between first and third cohort group means were tested with t-test. Categorical variables were described with absolute and relative frequencies by cohort. Cohort differences were tested with Pearson’s Chi-squared test.

To compare the differences between cohorts in change in gait speed and chair stands, linear mixed models were used. As study participants were measured multiple times, an individual-specific random intercept was added to models to take into account the correlation between observations. A linear mixed model approach was taken as it utilizes all the available data in parameter estimation. Physical performance variables were log-transformed because while examining the diagnostic plots with the untransformed outcome variables, the use of log-transformed variables provided better model fit (see Supplementary Fig. S1A-D). Estimation was performed with the REML (Restricted Maximum Likelihood) method to reduce bias in the standard errors of regression coefficients (Fitzmaurice et al. 2012), except when comparing models with a varying fixed effect part in which case models were estimated with the ML (Maximum Likelihood) method. Years since baseline as a continuous variable was used as the time variable. As the main interest of the study was in cohort differences in change in physical performance over time, the interaction term of time and cohort was included in all models. Separate models were fitted for men and women as there are gender differences both in the nature of the work careers (Nilsen et al. 2017) and in physical performance in older age (Wheaton and Crimmins 2016). First, the crude models including cohort, time and their interaction without covariates were fitted. Secondly, in model 2, we adjusted for baseline age and education, and finally in model 3 also for BMI, alcohol use, smoking and weekly total physical activity. Additionally, work status was added to the second and third model as a time-variant variable. Continuous variables were centred at their sample means. Finally, separate models for each psychosocial and physical job demand variable were fitted. The demand variables were added to the adjusted models including their two-way interactions with time and cohort. Also, three-way interactions between job demand, time and cohort were tested using F-tests and added to the models when significant. A sensitivity analysis was performed using only participants who at baseline worked 10 or more hours/week (N = 869). The significance level used was 0.05 at all steps. All analyses were conducted with R Software (R Core Team 2018) using packages lme4 (Bates et al. 2015) and lmerTest (Kuznetsova et al. 2017).

## Results

Characteristics of the participants at each cohort’s baseline are presented in Table 1. Psychosocial job demands increased for both women and men whereas physical job demands decreased across the cohorts. Baseline gait speed increased from cohort 1 to cohort 3 (0.99 m/s, SD 0.25 to 1.06 m/s, SD 0.23 for women and 0.99 m/s, SD 0.26 to 1.09 m/s, SD 0.23 for men, respectively). Conversely, chair stand performance decreased for both women and men. The correlation between gait speed and chair stand performance was 0.36 (women) and 0.33 (men) in the cohorts combined.

**Table 1:** Cohort differences at respective baselines for LASA cohorts measured in 1992–93 (Cohort 1), 2002–03 (Cohort 2) and 2012–13 (Cohort 3)

Women	Cohort 1	Cohort 2	Cohort 3	p
	n = 86	n = 139	n = 232	
Age, mean (standard deviation [SD])	59.2 (2.6)	58.5 (2.6)	59.2 (2.7)	0.998
Body mass index, mean (SD)	26.8 (3.8)	26.9 (4.6)	26.2 (4.6)	0.252
Working hours per week, mean (SD)	19.0 (16.0)	22.5 (14.9)	22.8 (11.0)	**0.044**
Total physical activity, MET hrs/week, mean (SD)	90.3 (47.9)	68.7 (43.2)	71.4 (47.2)	**0.002**
Alcohol use, n (%)				0.270
None	8 (9.3)	13 (9.4)	25 (10.8)	
Moderate	62 (72.1)	85 (61.2)	159 (68.5)	
High	16 (18.6)	41 (29.5)	48 (20.7)	
Smoking, n (%)				** <0.001**
Never	22 (25.6)	40 (28.8)	32 (13.8)	
Former	31 (36.0)	63 (45.3)	134 (57.8)	
Current	33 (38.4)	36 (25.9)	66 (28.4)	
Educational level, n (%)				** <0.001**
Low	28 (32.6)	20 (14.4)	15 (6.5)	
Moderate	42 (48.8)	89 (64.0)	142 (61.2)	
High	16 (18.6)	30 (21.6)	75 (32.3)	
Psychosocial job demands, n (%)				
Cognitive demands, high	16 (18.6)	40 (28.8)	82 (35.3)	**0.014**
Time pressure, high	18 (20.9)	42 (30.2)	96 (41.4)	**0.001**
Physical job demands, n (%)				
Use of force high	46 (53.5)	58 (41.7)	81 (34.9)	**0.010**
Repetitive moves high	64 (74.4)	97 (69.8)	122 (52.6)	** <0.001**
Gait speed, m/s, mean (SD)	0.99 (0.27)	0.98 (0.21)	1.08 (0.21)	**0.006**
Chair stand rise, times/s, mean (SD)	0.49 (0.11)	0.48 (0.12)	0.45 (0.10)	**0.001**

Men	n = 135	n = 219	n = 282	P
Age, mean (SD)	59.0 (2.8)	58.7 (2.6)	59.6 (2.6)	**0.030**
BMI, mean (SD)	26.1 (2.6)	27.3 (3.3)	27.4 (3.8)	** <0.001**
Working hours per week, mean (SD)	41.2 (15.8)	35.3 (15.8)	37.0 (13.2)	**0.007**
Total physical activity, MET hours/week	49.6 (41.1)	51.5 (44.4)	49.9 (40.5)	0.935
Alcohol use, n (%)				0.056
None	9 (6.7)	5 (2.3)	21 (7.4)	
Moderate	109 (80.7)	178 (81.3)	230 (81.6)	
High	17 (12.6)	36 (16.4)	31 (11.0)	
Smoking, n (%)				** <0.001**
Never	55 (40.7)	74 (33.8)	53 (18.8)	
Former	66 (48.9)	108 (49.3)	161 (57.1)	
Current	14 (10.4)	37 (16.9)	68 (24.1)	
Educational level, n (%)				** <0.001**
Low	16 (11.9)	33 (15.1)	16 (5.7)	
Moderate	90 (66.7)	104 (47.5)	149 (52.8)	
High	29 (21.5)	82 (37.4)	117 (41.5)	
Psychosocial job demands, n (%)				
Cognitive demands, high	36 (26.7)	76 (34.7)	144 (51.1)	** <0.001**
Time pressure, high	40 (29.6)	83 (37.9)	155 (55.0)	** <0.001**
Physical job demands, n (%)				
Use of force, high	63 (46.7)	105 (47.9)	109 (38.7)	0.082
Repetitive moves, high	93 (68.9)	131 (59.8)	123 (43.6)	** <0.001**
Gait speed, m/s, mean (SD)	1.02 (0.23)	1.06 (0.29)	1.09 (0.23)	**0.002**
Chair stand rise, times/s, mean (SD)	0.52 (0.13)	0.48 (0.13)	0.44 (0.10)	** <0.001**

Values in bold are statistically significant at alpha = 0.05

MET = metabolic equivalent of task based on hours/week spent on each activity, BMI = body mass index

### Cohort differences in physical performance

Cohort differences at baseline and across the 6-year follow-up for gait speed (m/s) and chair stand (times/s) are presented in Table 2. Women in cohort 3 had 8.1% (p = 0.003) and men in cohorts 2 and 3 had respectively 4.8% (p = 0.047) and 8.3% (p < 0.001) faster *gait speed* at baseline compared to women and men in cohort 1. After adjustments, the associations attenuated and were non-significant, however, for women in cohort 3 the association was borderline significant (5.7% faster gait speed, p = 0.051 compared to cohort 1). During the follow-up, gait speed of women in the first cohort decreased by 1.1% annually, but adjustments attenuated this decrease. Among women, the rate of change in gait speed in cohort 2 was more positive by an average of 1.3 percentage point (pp) annually compared to cohort 1 (p = 0.047) and attenuated after adjustments only slightly (p = 0.051).

**Table 2:** Differences for gait speed (m/s) and chair stand rise (times/s) at baseline and across 6-year follow-ups in three LASA cohorts measured in 1992–93 (cohort 1, ref.), 2002–03 (cohort 2) and 2012–13 (cohort 3)

	Women			Men		
	Model 1^a^	Model 2^b^	Model 3^c^	Model 1^a^	Model 2^b^	Model 3^c^
	B (95% CI)	B (95% CI)	B (95% CI)	B (95% CI)	B (95% CI)	B (95% CI)
*Gait speed* (m/s)						
Constant	−0.040 (−0.085, 0.005)	−0.112 (−0.175, −0.048)	−0.229 (−0.316, −0.141)	−0.022 (−0.059, 0.015)	−0.160 (−0.220, −0.100)	−0.212 (−0.297, −0.126)
Cohort 2	0.001 (−0.057, 0.059)	−0.019 (−0.077, 0.039)	−0.012 (−0.072, 0.048)	**0.048 (0.001, 0.096)**	0.039 (−0.008, 0.086)	0.011 (−0.039, 0.060)
Cohort 3	**0.081 (0.028, 0.133)**	**0.055 (0.001, 0.108)**	0.057 (0.000, 0.113)	**0.083 (0.037, 0.128)**	**0.072 (0.027, 0.117)**	0.045 (−0.003, 0.093)
Time	**−0.011 (−0.020, −0.001)**	−0.010 (−0.020, 0.000)	−0.011 (−0.022, 0.001)	−0.006 (−0.014, 0.002)	−0.001 (−0.010, 0.008)	−0.005 (−0.014, 0.005)
Cohort 2*time	**0.013 (0.000, 0.025)**	**0.013 (0.000, 0.025)**	0.013 (−0.000, 0.026)	0.003 (−0.008, 0.013)	0.001 (−0.009, 0.011)	0.005 (−0.006, 0.016)
Cohort 3*time	−0.001 (−0.012, 0.011)	−0.001 (−0.012, 0.011)	−0.001 (−0.013, 0.011)	−0.007 (−0.017, 0.003)	−0.010 (−0.020, 0.001)	−0.006 (−0.017, 0.005)
*Chair stand rise* (times/s)						
Constant	−0.730 (−0.777, −0.682)	−0.773 (−0.840, −0.706)	−0.899 (−0.994, −0.805)	−0.711 (−0.750, −0.672)	−0.753 (−0.818, −0.687)	−0.802 (−0.894, −0.710)
Cohort 2	−0.035 (−0.095, 0.026)	−0.047 (−0.108, 0.014)	−0.033 (−0.096, 0.030)	−0.036 (−0.086, 0.015)	−0.041 (−0.092, 0.009)	**−0.068 (−0.120, −0.017)**
Cohort 3	**−0.097 (−0.153, −0.042)**	**−0.110 (−0.167, −0.053)**	**−0.101 (−0.161, −0.041)**	**−0.148 (−0.196, −0.100)**	**−0.155 (−0.204, −0.107)**	**−0.165 (−0.215, −0.115)**
Time	**−0.010 (−0.019, −0.000)**	−0.009 (−0.019, 0.001)	−0.010 (−0.021, 0.001)	**−0.014 (−0.022, −0.006)**	**−0.012 (−0.021, −0.003)**	**−0.011 (−0.020, −0.002)**
Cohort 2*time	0.008 (−0.004, 0.020)	0.008 (−0.004, 0.020)	0.009 (−0.003, 0.022)	0.009 (−0.001, 0.019)	0.009 (−0.002, 0.019)	0.007 (−0.003, 0.017)
Cohort 3*time	0.010 (−0.001, 0.021)	0.010 (−0.002, 0.021)	0.011 (−0.002, 0.023)	**0.016 (0.006, 0.026)**	**0.015 (0.005, 0.025)**	**0.012 (0.002, 0.022)**

^a^Crude model

^b^Adjusted for age at baseline, education and work status

^c^Adjusted for age at baseline, education, work status, BMI, smoking, alcohol use and total physical activity

Total physical activity MET based on hours/week spent on each activity divided by ten, where the coefficients refer to the change in ten MET

Coefficients in bold are statistically significant at alpha = 0.05

Regarding *chair stand performance*, both women (9.7%, p = 0.001) and men (14.8%, p < 0.001) in cohort 3 performed worse at baseline compared to cohort 1 (Table 2). This association remained statistically significant after adjustments, 10.1% (p = 0.001) and 16.5% (p < 0.001) for women and men respectively. During the follow-up, the ability to perform chair stands in the first cohort decreased by 1.0% (p = 0.040) in women and 1.4% (p < 0.001) in men annually. After adjustments, this remained statistically significant only in men (1.1%, p = 0.013). Among men, after adjustments, in cohort 3 chair stand performance decreased less by an average of about 1.2 pp annually compared to cohort 1 (p = 0.016). There were no such associations found among women.

### Differences in physical performance according to job demands

**Fig. 1:**
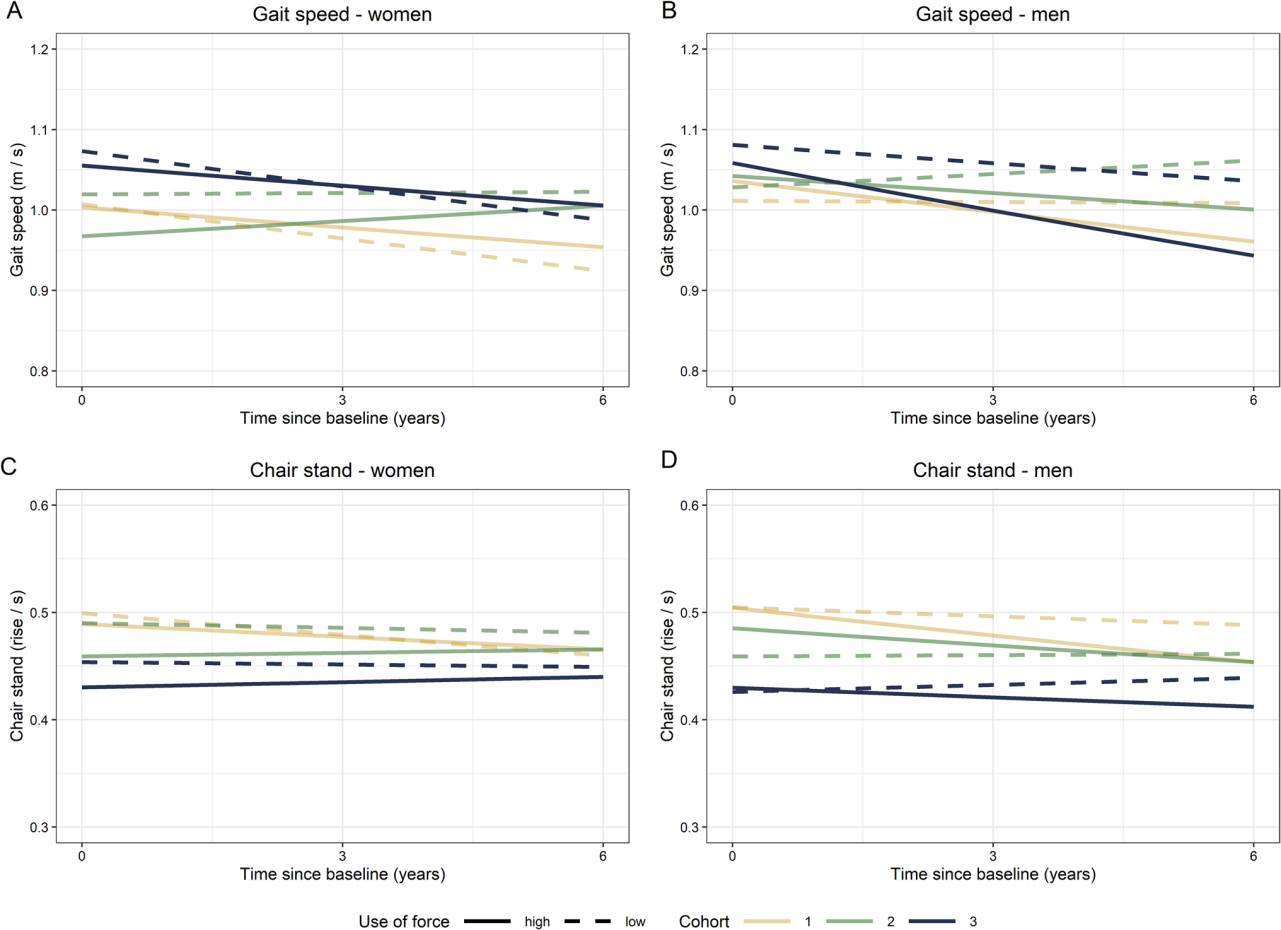
Change in gait speed and chair stands according to use of force in three cohorts, women and men. Covariates were kept at their means or the most frequent class. Plots are based on back-transformations of linear mixed models including use of force. High and low use of force-groups are presented by solid and dashed lines, respectively. Yellow, green and blue refer to Cohorts 1, 2 and 3, respectively

No between cohort differences were found in women or men in the associations between baseline physical or psychosocial job demands and 6-year follow-up of physical performance across the three cohorts assessed 10 years apart (Supplementary Table S2). The associations between job demands at baseline and change in physical performance are presented in Table 3. For men, the interaction between time and use of force in the fully adjusted model suggested a 1.2 pp faster decline in gait speed when comparing higher and lower use of force keeping other variables fixed (p = 0.005) (Fig. 1B). Greater use of force (Fig. 1D) and repetitive movements were associated with a 1.2 pp (p = 0.002) and 0.9 pp (p = 0.021) faster decline in chair stand performance, respectively. For women, regardless of the cohort, no interactions between time and job demands in gait speed or chair stand test were observed (as presented for use of force in Fig. 1A and C).

**Table 3:** Effects of job demands on gait speed (m/s) and chair stand (times/s) with job demands, 3-way interactions included, added to the fully adjusted model

Variable	Physical job demands		Psychosocial job demands	
	Use of force	Repetitive movements	Time pressure	Cognitive demands
	B (95% CI)^a^	B (95% CI)^a^	B (95% CI)^a^	B (95% CI)^a^
*Gait speed—women*				
Cohort 2	0.012 (−0.063, 0.087)	0.012 (−0.083, 0.108)	0.003 (−0.063, 0.069)	0.003 (−0.062, 0.068)
Cohort 3	0.064 (−0.006, 0.134)	0.064 (−0.023, 0.151)	0.062 (−0.001, 0.125)	0.060 (−0.003, 0.122)
Time	**−0.014 (−0.027, −0.002)**	−0.012 (−0.025, 0.001)	**−0.011 (−0.023, −0.000)**	**−0.012 (−0.023, −0.001)**
Job demand	−0.004 (−0.084, 0.076)	−0.005 (−0.096, 0.087)	0.046 (−0.055, 0.147)	0.019 (−0.084, 0.123)
Cohort 2*time	**0.015 (0.002, 0.028)**	**0.014 (0.001, 0.027)**	**0.014 (0.000, 0.027)**	**0.013 (0.000, 0.027)**
Cohort 3*time	0.000 (−0.012, 0.013)	−0.001 (−0.013, 0.012)	−0.001 (−0.014, 0.011)	−0.002 (−0.014, 0.011)
Cohort 2*job demand	−0.049 (−0.142, 0.045)	−0.029 (−0.133, 0.075)	−0.044 (−0.153, 0.066)	−0.043 (−0.156, 0.070)
Cohort 3*job demand	−0.013 (−0.102, 0.075)	−0.006 (−0.103, 0.091)	−0.018 (−0.122, 0.085)	−0.005 (−0.111, 0.102)
Time*job demand	0.006 (−0.004, 0.015)	0.001 (−0.009, 0.011)	0.003 (−0.007, 0.013)	0.006 (−0.005, 0.016)
*Gait speed—men*				
Cohort 2	0.016 (−0.046, 0.078)	0.022 (−0.052, 0.096)	0.018 (−0.038, 0.074)	0.020 (−0.036, 0.075)
Cohort 3	**0.066 (0.007, 0.126)**	0.068 (−0.002, 0.138)	0.044 (−0.013, 0.101)	0.038 (−0.018, 0.093)
Time	−0.000 (−0.011, 0.010)	−0.000 (−0.012, 0.011)	−0.008 (−0.017, 0.002)	−0.007 (−0.017, 0.003)
Job demand	0.024 (−0.040, 0.088)	0.003 (−0.068, 0.073)	0.038 (−0.034, 0.109)	0.040 (−0.034, 0.114)
Cohort 2*time	0.006 (−0.005, 0.017)	0.005 (−0.006, 0.016)	0.005 (−0.006, 0.016)	0.005 (−0.006, 0.017)
Cohort 3*time	−0.007 (−0.018, 0.004)	−0.008 (−0.019, 0.004)	−0.007 (−0.018, 0.004)	−0.006 (−0.018, 0.005)
Cohort 2*job demand	−0.011 (−0.087, 0.066)	−0.015 (−0.096, 0.067)	−0.024 (−0.107, 0.059)	−0.033 (−0.119, 0.053)
Cohort 3*job demand	−0.045 (−0.120, 0.030)	−0.041 (−0.120, 0.038)	−0.011 (−0.090, 0.069)	−0.002 (−0.083, 0.079)
Time*job demand	**−0.012 (−0.021, −0.004)**	−0.008 (−0.017, 0.000)	0.005 (−0.003, 0.014)	0.004 (−0.004, 0.013)
*Chair stand rise—women*				
Cohort 2	−0.018 (−0.099, 0.062)	0.007 (−0.099, 0.113)	−0.043 (−0.114, 0.027)	−0.034 (−0.104, 0.035)
Cohort 3	**−0.096 (−0.172, −0.020)**	−0.074 (−0.171, 0.023)	**−0.119 (−0.187, −0.050)**	**−0.112 (−0.179, −0.045)**
Time	**−0.014 (−0.026, −0.002)**	**−0.013 (−0.026, −0.001)**	−0.011 (−0.022, 0.000)	**−0.011 (−0.022, −0.000)**
Job demand	−0.020 (−0.109, 0.068)	0.036 (−0.067, 0.138)	−0.050 (−0.164, 0.064)	−0.045 (−0.162, 0.072)
Cohort 2*time	0.011 (−0.002, 0.023)	0.010 (−0.003, 0.023)	0.010 (−0.003, 0.023)	0.010 (−0.003, 0.023)
Cohort 3*time	0.012 (−0.000, 0.024)	0.012 (−0.001, 0.024)	0.011 (−0.001, 0.023)	0.011 (−0.001, 0.023)
Cohort 2*job demand	−0.045 (−0.150, 0.060)	−0.061 (−0.178, 0.056)	0.032 (−0.092, 0.156)	0.001 (−0.127, 0.129)
Cohort 3*job demand	−0.033 (−0.132, 0.066)	−0.046 (−0.155, 0.063)	0.050 (−0.067, 0.167)	0.028 (−0.093, 0.149)
Time*job demand	0.005 (−0.004, 0.015)	0.004 (−0.006, 0.013)	0.001 (−0.009, 0.010)	0.002 (−0.008, 0.012)
*Chair stand rise—men*				
Cohort 2	**−0.095 (−0.162, −0.029)**	−0.073 (−0.153, 0.006)	−0.051 (−0.111, 0.009)	−0.052 (−0.111, 0.007)
Cohort 3	**−0.170 (−0.234, −0.106)**	**−0.165 (−0.241, −0.090)**	**−0.148 (−0.209, −0.088)**	**−0.154 (−0.213, −0.095)**
Time	−0.006 (−0.015, 0.004)	−0.005 (−0.015, 0.005)	−0.013 (−0.023, −0.004)	−0.012 (−0.021, −0.002)
Job demand	−0.001 (−0.071, 0.070)	0.008 (−0.070, 0.085)	0.022 (−0.057, 0.100)	0.073 (−0.008, 0.154)
Cohort 2*time	0.006 (−0.004, 0.017)	0.006 (−0.005, 0.016)	0.006 (−0.005, 0.016)	0.006 (−0.004, 0.017)
Cohort 3*time	**0.011 (0.001, 0.021)**	0.010 (−0.001, 0.020)	0.010 (−0.000, 0.020)	**0.012 (0.001, 0.022)**
Cohort 2*job demand	0.056 (−0.029, 0.141)	0.012 (−0.080, 0.103)	−0.049 (−0.141, 0.043)	−0.056 (−0.151, 0.039)
Cohort 3*job demand	0.010 (−0.074, 0.094)	0.005 (−0.083, 0.094)	−0.041 (−0.131, 0.047)	−0.055 (−0.145, 0.036)
Time*job demand	**−0.012 (−0.020, −0.004)**	**−0.009 (−0.017, −0.001)**	0.007 (−0.001, 0.015)	0.002 (−0.006, 0.010)

^a^Adjusted for age at baseline, education, work status, BMI, smoking, alcohol use and total physical activity

Total physical activity MET based on hours/week spent on each activity divided by ten, where the coefficients refer to the change in ten MET

Coefficients in bold are statistically significant at alpha = 0.05

The sensitivity analyses, conducted for the participants who worked at least 10 h per week, resulted in associations in line with those that included participants who reported working at least 1 h per week, see Supplementary Tables S3–S5.

## Discussion

To the best of our knowledge, this is the first study to investigate cohort differences in the association between physical and psychological job demands and change in physical performance among older employees. To do so, we compared three independent population-based samples of workers aged 55 to 65 with baseline data on job demands in 1992–1993, 2002–2003 and 2012–2013, respectively and physical performance over a six-year period. No between cohort differences for men and women were found for the association between job demands and changes in physical performance across the cohorts. Higher physical job demands affected the six-year rate of change in physical performance negatively among men but not women. Such associations were not observed for psychosocial job demands and physical performance after controlling for confounders. Furthermore, we found that the most recent cohort had faster gait speed at baseline, but slower chair stand speed compared to their counterparts measured two decades earlier. As we are dealing with samples of workers, we might look for an explanation in terms of work exposures. Gait speed has been shown to be a good indicator of vitality (Studenski et al. 2011 JAMA). The increase in gait speed may then be explained by the lower prevalence of high physical work demands which may lead to better health in older workers. In contrast, the prevalence of sedentary jobs has increased, which may have led to a worse lower-body strength as indicated by the chair stands test.

Note, that the association for gait speed attenuated somewhat after accounting for confounders.

Against our expectations, we observed no cohort differences over the past 20 years in the association between physical work demands and change in physical performance. This suggests that the negative effect of work demands has not changed. Regarding physical job demands, we found that work that required higher use of force and/or included repetitive movements was associated with faster decline in gait speed and in chair stand performance in men. Albeit the research evidence on the association between physical job demands and objectively measured physical performance have been inconsistent (Torgén et al. 1999; van der Noordt et al. 2019), our findings support the study by Møller et al., which reported a negative association between physical job demands and chair stand performance among middle-aged men but not women (Møller et al. 2015). These findings may be explained by greater wear and tear of the body as a consequence of exposure to physical job demands. Performing the chair stand test requires adequate muscle power in the lower extremities, which is known to decrease with older age (Larsson et al. 1979). Thus, faster decline in the chair stand test performance may be an indicator of accelerated decline in musculoskeletal health exacerbated by higher physical job demands (Gerr et al. 2014). Similar to the study by Møller et al. (2015), we did not find this association for women. This might be due to the preponderance of part-time work in Dutch women, resulting in a shorter weekly duration of exposure to physical job demands compared to men. However, little is known about the gender differences in the relation between work exposures and musculoskeletal aging across working life (Møller et al. 2015).

Regarding psychosocial job demands, the proportion of employees with higher demands has increased significantly over the past decades (Gallie 2005), which was also evident in the present study. Again, there were no cohort differences during the 6-year follow-up for the association between psychological work demands and change in physical performance. Furthermore, we did not find any association between psychosocial job demands and the rate of change in the physical performance measures. In line with our findings, a Swedish study found no association between high job demands in late midlife and physical performance measure scores in old age (Nilsen et al. 2017). Studies on the association between psychological job demands and self-rated physical functioning have been inconsistent for men and women. A US study found that lower psychological job demand were related to a better score on the physical functioning sub-scale of Short Form-36 health survey among middle-aged female nurses (Cheng et al. 2000). In the Whitehall II Study, high psychological job demands increased the odds for poor physical functioning among women but not men (Stansfeld et al. 1998). In a UK birth cohort, no associations were found between psychological job demands and the SF-36 physical summary component (von Bonsdorff et al. 2014). A Danish study found that men who often reported high work pace had a higher risk of mobility limitations while for women, reporting high work pace often protected from mobility limitations (Hansen et al. 2014). All in all, these differences may be due to differences in the measurements used for assessing physical functioning/performance as well as differences in work context in the different countries.

### Strengths and weaknesses

One of the main strengths of this study was that it was based on a nationally representative population-based dataset. The first LASA cohort was studied first in 1992 and currently the dataset includes three birth cohorts, each with multiple follow-up waves. This provided a unique opportunity to compare physical performance according to job demands of the older working population across three decades using the same standardized measurement instruments. This study also had some limitations. First, the GPJEM does not take into account heterogeneity within job categories, because job demand information is aggregated (Rijs et al. 2014). Second, the ‘healthy worker effect’ might have influenced our findings because information on the current job was used, as opposed to the longest held job. Employees with reduced functioning may have switched to less demanding work because of not being able to continue to work in more demanding jobs. This may have limited our ability to detect relevant associations. However, data on the longest held job was available for cohort 1 showing that only a small minority of the respondents reported a different longest held job compared to the current job (Deeg et al. 2021) and the associated working conditions remained on average the same, thus proving some evidence for a lesser healthy worker effect in our data. However, in recent years changing jobs has been more frequent than it used to be.

## Conclusions

This study showed that job demands were similarly associated with physical performance over six years across three cohorts. Regardless of the cohort, higher physical job demands of older employees aged 55 to 65 were associated with stronger six-year rates of decline in physical performance in men, while no associations were found among women. Furthermore, no associations were found between psychosocial job demands and change in physical performance in the cohorts. This could suggest that physical job demands have a lasting impact on physical performance in older age, particularly among men and that this situation has not appeared to improve in the past decades. Since nowadays more older workers need to continue working up to higher ages, it is important to alleviate their working conditions. If not, the health of more older workers would be affected in the long term, which would present an extra burden on health care and society. Work wellbeing interventions should primarily be focused on employees working in jobs that include high physical job demands.

## Supplementary Information

Below is the link to the electronic supplementary material.

Supplementary materials (228 KB)

## Data Availability

The datasets generated and analysed during the current study are not publicly available due the fact that they constitute an excerpt of research in progress but are available from the corresponding author on reasonable request.
