# Supplementary material for: Changes in physical performance according to job demands across three cohorts of older workers in the Longitudinal Aging Study Amsterdam
Source: Eur J Ageing. 2023 Jun 7;20(1):21. doi: 10.1007/s10433-023-00768-9 (PMC10247630; doi:10.1007/s10433-023-00768-9)
Supplement: Supplementary file 1 — Supplementary materials (228 KB) [file 10433_2023_768_MOESM1_ESM.docx]

**SUPPLEMENTARY MATERIAL**

Table S1. Drop-out and item-non-response in the three cohorts during the three time points.

| **Cohort** | **Time^a^** | **In Sample^b^** | **Drop-out^c^** | **Item-non-response^d^** | **Included^e^** |
| --- | --- | --- | --- | --- | --- |
|  |  |  |  |  |  |
| 1 | 1 | 274 | 0 | 53 | 221 |
| 1 | 2 | 242 | 32 | 42 | 200 |
| 1 | 3 | 224 | 18 | 38 | 186 |
| 2 | 1 | 416 | 0 | 58 | 358 |
| 2 | 2 | 377 | 39 | 40 | 337 |
| 2 | 3 | 345 | 32 | 41 | 304 |
| 3 | 1 | 618 | 0 | 104 | 514 |
| 3 | 2 | 474 | 144 | 64 | 410 |
| 3 | 3 | 406 | 68 | 47 | 359 |
| **Pooled cohorts** |  |  |  |  |  |
|  | 1 | 1308 | 0 | 215 | 1093 |
|  | 2 | 1093 | 215 | 146 | 947 |
|  | 3 | 975 | 118 | 126 | 849 |

^a^Time: 1= baselines, 2=3-year follow-ups, 3=6-year follow-ups for the three LASA cohorts

^b^In sample: number of individuals included in the sample at a certain time point

^c^Drop-out: number of individuals who dropped out at a certain time point

^d^Item-non-response: number of individuals out of those who had not dropped out, who had missing values at least in one of the variables used for the adjusted models in table 2.

^e^Included: number of individuals who contributed to parameter estimation of adjusted models in table 2

Table S2. F-tests p-values for the three-way interactions for physical or psychosocial job demands and 6-year follow-up of physical performance across the three cohorts

|  | **Use of force** | **Repetitive movements** | **Time pressure** | **Cognitive demands** |
| --- | --- | --- | --- | --- |
| **Women** |  |  |  |  |
| Gait speed | 0.687 | 0.288 | 0.627 | 0.518 |
| Chair stand rise | 0.352 | 0.580 | 0.659 | 0.760 |
| **Men** |  |  |  |  |
| Gait speed | 0.355 | 0.464 | 0.459 | 0.958 |
| Chair stand rise | 0.574 | 0.918 | 0.611 | 0.895 |

Table S3. Cohort differences at respective baselines for LASA cohorts measured in 1992-93 (Cohort 1), 2002-03 (Cohort 2) and 2012-13 (Cohort 3) for the participants who worked at least 10 hours/week.

|  | **Cohort 1** | **Cohort 2** | **Cohort 3** | **p** |
| --- | --- | --- | --- | --- |
| **Women** | **n=54** | **n=109** | **n=204** |  |
| Age, mean (SD) | 58.7 (2.4) | 58.2 (2.5) | 59.1 (2.6) | .214 |
| BMI, mean (SD) | 26.8 (3.9) | 27.1 (4.6) | 26.1 (4.7) | .276 |
| Working hours per week, mean (SD) | 26.1 (15.9) | 27.0 (13.4) | 25.2 (9.5) | .669 |
| Total physical activity, MET hrs/week, mean (SD) | 85.8 (46.5) | 67.0 (44.8) | 69.1 (48.7) | **.023** |
| Alcohol use, n (%) |  |  |  | .321 |
| None | 7 (13.0) | 10 (9.2) | 20 (9.8) |  |
| Moderate | 39 (72.2) | 67 (61.5) | 138 (67.6) |  |
| High | 8 (14.8) | 32 (29.4) | 46 (22.5) |  |
| Smoking, n (%) |  |  |  | **<.001** |
| Never | 17 (31.5) | 34 (31.2) | 29 (14.2) |  |
| Former | 19 (35.2) | 52 (47.7) | 115 (56.4) |  |
| Current | 18 (33.3) | 23 (21.1) | 60 (29.4) |  |
| Educational level, n (%) |  |  |  | **.001** |
| Low | 13 (24.1) | 15 (13.8) | 13 (6.4) |  |
| Moderate | 29 (53.7) | 69 (63.3) | 121 (59.3) |  |
| High | 12 (22.2) | 25 (22.9) | 70 (34.3) |  |
| Psychosocial job demands, n (%) |  |  |  |  |
| Cognitive demands, high | 11 (20.4) | 35 (32.1) | 75 (36.8) | .073 |
| Time pressure, high | 13 (24.1) | 37 (33.9) | 90 (44.1) | **.015** |
| Physical job demands, n (%) |  |  |  |  |
| Use of force high | 24 (44.4) | 38 (34.9) | 64 (31.4) | .196 |
| Repetitive moves high | 39 (72.2) | 72 (66.1) | 101 (49.5) | **.001** |
| Gait speed, m/s, mean (SD) | 1.02 (0.30) | 1.00 (0.20) | 1.08 (0.21) | .197 |
| Chair stand rise, times/s, mean (SD) | 0.48 (0.12) | 0.49 (0.12) | 0.45 (0.10) | **.036** |
| **Men** | **n=131** | **n=201** | **n=270** |  |
| Age, mean (SD) | 58.9 (2.8) | 58.5 (2.5) | 59.5 (2.6) | .070 |
| BMI, mean (SD) | 26.2 (2.6) | 27.3 (3.3) | 27.5 (3.8) | **<.001** |
| Working hours per week, mean (SD) | 42.0 (15.0) | 37.9 (13.6) | 38.2 (11.9) | **.013** |
| Total physical activity, MET hours/week | 47.7 (34.6) | 52.0 (45.4) | 48.7 (38.7) | .801 |
| Alcohol use, n (%) |  |  |  | .106 |
| None | 9 (6.9) | 5 (2.5) | 18 (6.7) |  |
| Moderate | 105 (80.2) | 161 (80.1) | 221 (81.9) |  |
| High | 17 (13.0) | 35 (17.4) | 31 (11.5) |  |
| Smoking, n (%) |  |  |  | **<.001** |
| Never | 52 (39.7) | 67 (33.3) | 53 (19.6) |  |
| Former | 65 (49.6) | 98 (48.8) | 152 (56.3) |  |
| Current | 14 (10.7) | 36 (17.9) | 65 (24.1) |  |
| Educational level, n (%) |  |  |  | **<.001** |
| Low | 15 (11.5) | 30 (14.9) | 16 (5.9) |  |
| Moderate | 87 (66.4) | 97 (48.3) | 141 (52.2) |  |
| High | 29 (22.1) | 74 (36.8) | 113 (41.9) |  |
| Psychosocial job demands, n (%) |  |  |  |  |
| Cognitive demands, high | 35 (26.7) | 67 (33.3) | 137 (50.7) | **<.001** |
| Time pressure, high | 39 (29.8) | 74 (36.8) | 151 (55.9) | **<.001** |
| Physical job demands, n (%) |  |  |  |  |
| Use of force, high | 61 (46.6) | 98 (48.8) | 104 (38.5) | .065 |
| Repetitive moves, high | 90 (68.7) | 121 (60.2) | 117 (43.3) | **<.001** |
| Gait speed, m/s, mean (SD) | 1.02 (0.24) | 1.07 (0.29) | 1.09 (0.23) | **.005** |
| Chair stand rise, times/s, mean (SD) | 0.52 (0.14) | 0.48 (0.13) | 0.44 (0.10) | **<.001** |

Table S4. Differences for gait speed (m/s) and chair stand rise (times/s) at baseline and across 6-year follow-ups in three LASA cohorts measured in 1992-93 (cohort 1, ref.), 2002-03 (cohort 2) and 2012-13 (cohort 3) for the participants who worked at least 10 hours/week.

|  | **Women** | | | **Men** | | |
| --- | --- | --- | --- | --- | --- | --- |
|  | **Model 1^a^** | **Model 2^b^** | **Model 3^c^** | **Model 1^a^** | **Model 2^b^** | **Model 3^c^** |
|  | **B (95% CI)** | **B (95% CI)** | **B (95% CI)** | **B (95% CI)** | **B (95% CI)** | **B (95% CI)** |
| **Gait speed (m/s)** | | | | | | |
| Constant | -0.028 (-0.081,0.025) | -0.111 (-0.184, -0.038) | -0.217 (-0.312, -0.122) | -0.019 (-0.057,0.018) | -0.158 (-0.221, -0.096) | -0.203 (-0292, -0.114) |
| Cohort 2 | 0.003 (-0.062, 0.069) | -0.012 (-0.076, 0.053) | -0.006 (-0.073, 0.060) | **0.050 (0.001, 0.099)** | 0.041 (-0.007, 0.089) | 0.013 (-0.038, 0.064) |
| Cohort 3 | **0.079 (0.020, 0.139)** | 0.057 (-0.002, 0.117) | 0.053 (-0.009, 0.116) | **0.080 (0.034, 0.127)** | **0.070 (0.024, 0.116**) | 0.044 (-0.005, 0.093) |
| Time | **-0.012 (-0.023, -0.001)** | **-0.013 (-0.025, -0.001)** | **-0.015 (-0.028, -0.003)** | -0.007 (-0.015, 0.002) | -0.001 (-0.010, 0.008) | -0.005 (-0.014, 0.005) |
| Cohort 2*time | **0.015 (0.001, 0.028)** | **0.016 (0.002, 0.029)** | **0.017 (0.003, 0.032)** | 0.003 (-0.007, 0.014) | 0.002 (-0.009, 0.013) | 0.006 (-0.005, 0.018) |
| Cohort 3*time | -0.001 (-0.013, 0.012) | 0.000 (-0.012, 0.013) | 0.000 (-0.013, 0.014) | -0.006 (-0.016, 0.004) | -0.009 (-0.019, 0.002) | -0.005 (-0.016, 0.006) |
| **Chair stand rise (times/s)** | | | | | | |
| Constant | -0.744 (-0.804, -0.684) | -0.796 (-0.879, -0.712) | -0.948 (-1.060, -0.837) | -0.703 (-0.743, -0.664) | -0.746 (-0.813, -0.679) | -0.796 (-0.892, -0.700) |
| Cohort 2 | -0.002 (-0.076, 0.071) | -0.009 (-0.083, 0.065) | 0.016 (-0.061, 0.093) | -0.042 (-0.093, 0.009) | -0.047 (-0.098, 0.005) | **-0.069 (-0.122, -0.016)** |
| Cohort 3 | **-0.081 (-0.149, -0.014)** | **-0.089 (-0.158, -0.020)** | **-0.082 (-0.155, -0.010)** | **-0.154 (-0.202, -0.105)** | **-0.159 (-0.208, -0.110)** | -**0.164 (-0.215, -0.113)** |
| Time | -0.005 (-0.017, 0.007) | -0.003 (-0.016, 0.010) | -0.005 (-0.019, 0.009) | **-0.016 (-0.024, -0.008)** | **-0.013 (-0.022, -0.004)** | **-0.012 (-0.020, -0.003)** |
| Cohort 2*time | 0.001 (-0.014, 0.016) | 0.000 (-0.014, 0.015) | 0.002 (-0.013, 0.018) | 0.011 (-0.000, 0.021) | 0.010 (-0.001, 0.021) | 0.008 (-0.002, 0.019) |
| Cohort 3*time | 0.005 (-0.008, 0.019) | 0.004 (-0.009, 0.018) | 0.006 (-0.009, 0.021) | **0.017 (0.007, 0.027)** | **0.016 (0.006, 0.026)** | **0.013 (0.002, 0.023)** |

^a^Crude model

^b^Adjusted for age at baseline, education and work status

^c^Adjusted for age at baseline, education, work status, BMI, smoking, alcohol use and total physical activity

Total physical activity MET based on hours/week spent on each activity divided by ten, where the coefficients refer to the change in ten MET

Coefficients in bold are statistically significant at alpha=0.05

Table S5. Effects of job demands on gait speed (m/s) and chair stand (times/s) with job demands, 2-way interactions included, added to the fully adjusted model for the participants who worked at least 10 hours/week.

|  | **Physical job demands** | | **Psychosocial job demands** | |
| --- | --- | --- | --- | --- |
| Variable | Use of force | Repetitive movements | Time pressure | Cognitive demands |
| **Gait speed - women** | **B (95% CI)** | **B (95% CI)** | **B (95% CI)** | **B (95% CI)** |
| Cohort 2 | 0.011 (-0.068, 0.090) | 0.022 (-0.081, 0.125) | 0.021 (-0.053, 0.095) | 0.020 (-0.053, 0.093) |
| Cohort 3 | 0.056 (-0.019, 0.130) | 0.062 (-0.033, 0.156) | **0.074 (0.004, 0.144)** | **0.069 (0.001, 0.138)** |
| Time | **-0.016 (-0.030, -0.003)** | **-0.016 (-0.031, -0.002)** | **-0.017 (-0.030, -0.004)** | **-0.017 (-0.030, -0.005)** |
| Job demand | 0.015 (-0.076, 0.106) | 0.019 (-0.082, 0.120) | 0.065 (-0.044, 0.173) | 0.039 (-0.074, 0.151) |
| Cohort 2*time | **0.019 (0.004, 0.033)** | **0.019 (0.004, 0.033)** | **0.018 (0.003, 0.032)** | **0.018 (0.003, 0.032)** |
| Cohort 3*time | 0.001 (-0.013, 0.015) | 0.001 (-0.013, 0.015) | -0.000 (-0.014, 0.013) | -0.000 (-0.014, 0.013) |
| Cohort 2*job demand | -0.031 (-0.137, 0.075) | -0.034 (-0.147, 0.079) | -0.080 (-0.197, 0.038) | -0.080 (-0.202, 0.042) |
| Cohort 3*job demand | 0.008 (-0.092, 0.108) | -0.002 (-0.108, 0.103) | -0.061 (-0.171, 0.049) | -0.048 (-0.163, 0.067) |
| Time*job demand | 0.001 (-0.009, 0.011) | 0.001 (-0.009, 0.011) | 0.005 (-0.005, 0.015) | 0.007 (-0.003,0.017) |
| **Gait speed - men** |  |  |  |  |
| Cohort 2 | 0.020 (-0.044, 0.085) | 0.030 (-0.046, 0.106) | 0.018 (-0.040, 0.076) | 0.020 (-0.038, 0.077) |
| Cohort 3 | **0.064 (0.002, 0.125)** | 0.067 (-0.005, 0.138) | 0.044 (-0.015, 0.102) | 0.039 (-0.018, 0.095) |
| Time | -0.001 (-0.011, 0.010) | -0.001 (-0.012, 0.011) | -0.008 (-0.018, 0.002) | -0.008 (-0.018, 0.002) |
| Job demand | 0.021 (-0.045, 0.087) | 0.004 (-0.068, 0.076) | 0.037 (-0.037, 0.110) | 0.037 (-0.039, 0.113) |
| Cohort 2*time | 0.007 (-0.004, 0.019) | 0.006 (-0.005, 0.018) | 0.007 (-0.005, 0.018) | 0.007 (-0.005, 0.018) |
| Cohort 3*time | -0.006 (-0.017, 0.006) | -0.007 (-0.018, 0.005) | -0.006 (-0.018, 0.005) | -0.006 (-0.017, 0.005) |
| Cohort 2*job demand | -0.015 (-0.094, 0.064) | -0.025 (-0.109, 0.059) | -0.018 (-0.103, 0.068) | -0.027 (-0.116, 0.063) |
| Cohort 3*job demand | -0.041 (-0.118, 0.037) | -0.041 (-0.122, 0.040) | -0.011 (-0.093, 0.071) | -0.004 (-0.087, 0.080) |
| Time*job demand | **-0.012 (-0.020, -0.003)** | -0.008 (-0.017, 0.001) | 0.006 (-0.003, 0.015) | 0.006 (-0.003, 0.015) |
| **Chair stand rise - women** | | | | |
| Cohort 2 | 0.004 (-0.090, 0.098) | 0.024 (-0.101, 0.150) | 0.015 (-0.072, 0.102) | 0.026 (-0.060, 0.111) |
| Cohort 3 | **-0.094 (-0.182, -0.006)** | -0.080 (-0.195, 0.035) | **-0.101 (-0.184, -0.018)** | **-0.093 (-0.174, -0.013)** |
| Time | -0.008 (-0.023, 0.007) | -0.010 (-0.026, 0.006) | -0.006 (-0.020, 0.009) | -0.006 (-0.020, 0.008) |
| Job demand | -0.054 (-0.165, 0.056) | 0.007 (-0.117, 0.130) | -0.038 (-0.171, 0.096) | -0.028 (-0.168, 0.111) |
| Cohort 2*time | 0.003 (-0.013, 0.019) | 0.003 (-0.013, 0.019) | 0.003 (-0.013, 0.019) | 0.003 (-0.013, 0.019) |
| Cohort 3*time | 0.007 (-0.008, 0.022) | 0.008 (-0.007, 0.023) | 0.007 (-0.008, 0.022) | 0.007 (-0.009, 0.022) |
| Cohort 2*job demand | 0.009 (-0.120, 0.138) | -0.020 (-0.159, 0.118) | -0.006 (-0.151, 0.139) | -0.044 (-0.195, 0.107) |
| Cohort 3*job demand | 0.000 (-0.121, 0.123) | -0.017 (-0.146, 0.112) | 0.038 (-0.098, 0.173) | 0.012 (-0.129, 0.154) |
| Time*job demand | 0.005 (-0.006, 0.016) | 0.007 (-0.004, 0.017) | -0.000 (-0.011, 0.011) | 0.001 (-0.010, 0.012) |
| **Chair stand rise - men** | | | | |
| Cohort 2 | **-0.095 (-0.164, -0.027)** | -0.071 (-0.153, 0.011) | -0.054 (-0.115, 0.008) | -0.054 (-0.115, 0.006) |
| Cohort 3 | **-0.171 (-0.236, -0.106)** | **-0.164 (-0.241, -0.088)** | **-0.143 (-0.205, -0.081)** | **-0.150 (-0.210, -0.089)** |
| Time | -0.006 (-0.016, 0.003) | -0.006 (-0.016, 0.005) | **0.014 (-0.023, -0.005)** | **-0.012 (-0.022, -0.003)** |
| Job demand | -0.008 (-0.080, 0.064) | 0.001 (-0.078, 0.081) | 0.022 (-0.058, 0.103) | 0.073 (-0.010, 0.155) |
| Cohort 2*time | 0.008 (-0.003, 0.018) | 0.007 (-0.003, 0.018) | 0.007 (-0.003, 0.018) | 0.008 (-0.003, 0.018) |
| Cohort 3*time | **0.011 (0.001, 0.021)** | 0.010 (-0.001, 0.020) | 0.010 (-0.001, 0.020) | **0.011 (0.001, 0.022)** |
| Cohort 2*job demand | 0.055 (-0.033, 0.143) | 0.006 (-0.088, 0.099) | -0.046 (-0.141, 0.050) | -0.052 (-0.151, 0.046) |
| Cohort 3*job demand | 0.014 (-0.072, 0.100) | 0.004 (-0.086, 0.095) | -0.050 (-0.141, 0.041) | -0.060 (-0.153, 0.032) |
| Time*job demand | **-0.011 (-0.019, -0.003)** | **-0.008 (-0.017, -0.000)** | **0.008 (0.000, 0.016)** | 0.004 (-0.005, 0.012) |

^a^Crude model

^b^Adjusted for age at baseline, education, work status, BMI, smoking, alcohol use and total physical activity

Total physical activity MET based on hours/week spent on each activity divided by ten, where the coefficients refer to the change in ten MET

Coefficients in bold are statistically significant at alpha=0.05

**Women Gait speed**


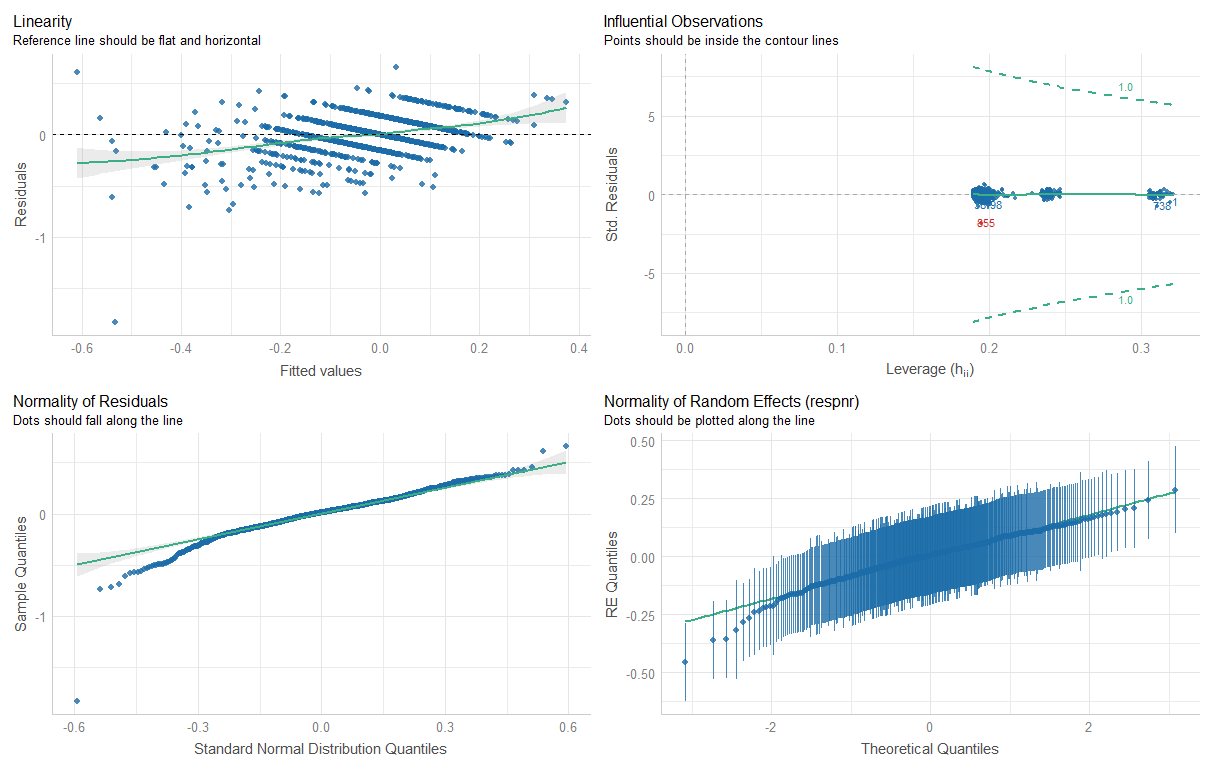


Figure S1A.

**Men Gait speed**


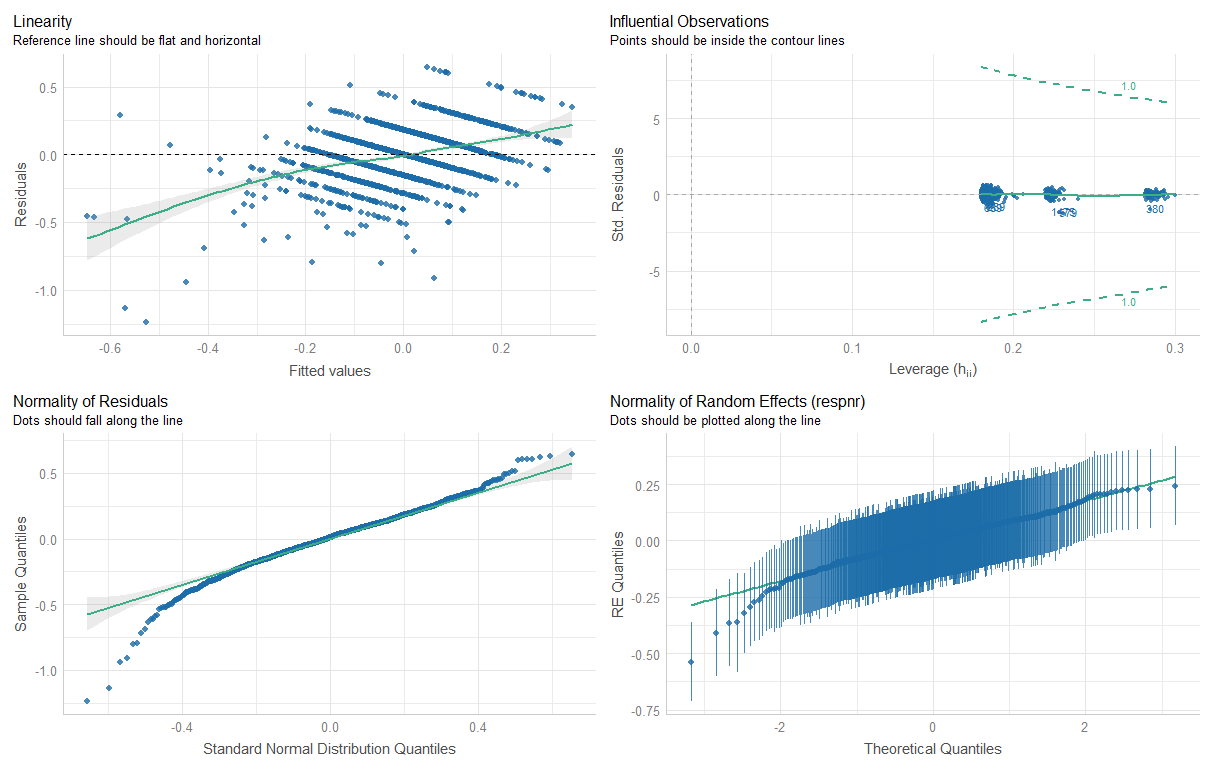


Figure S1B.

**Women Chair stand test**


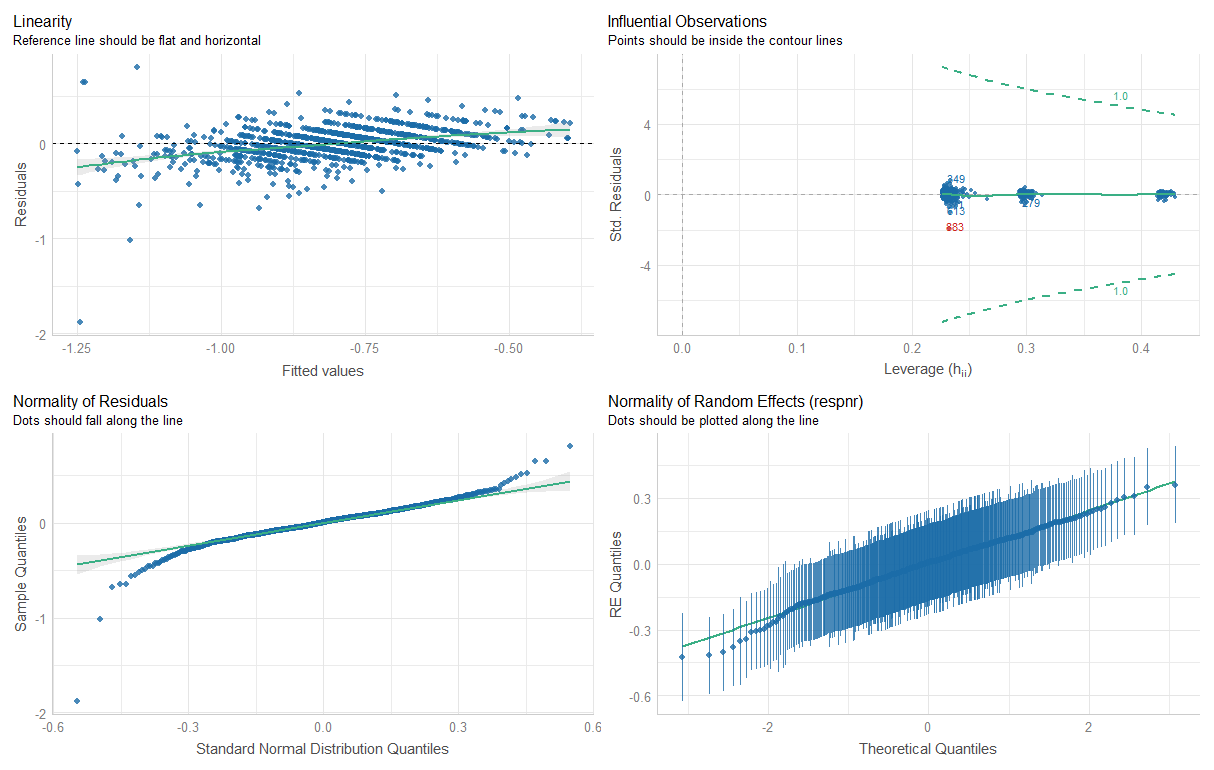


Figure S1C.

**Men Chair stand test**


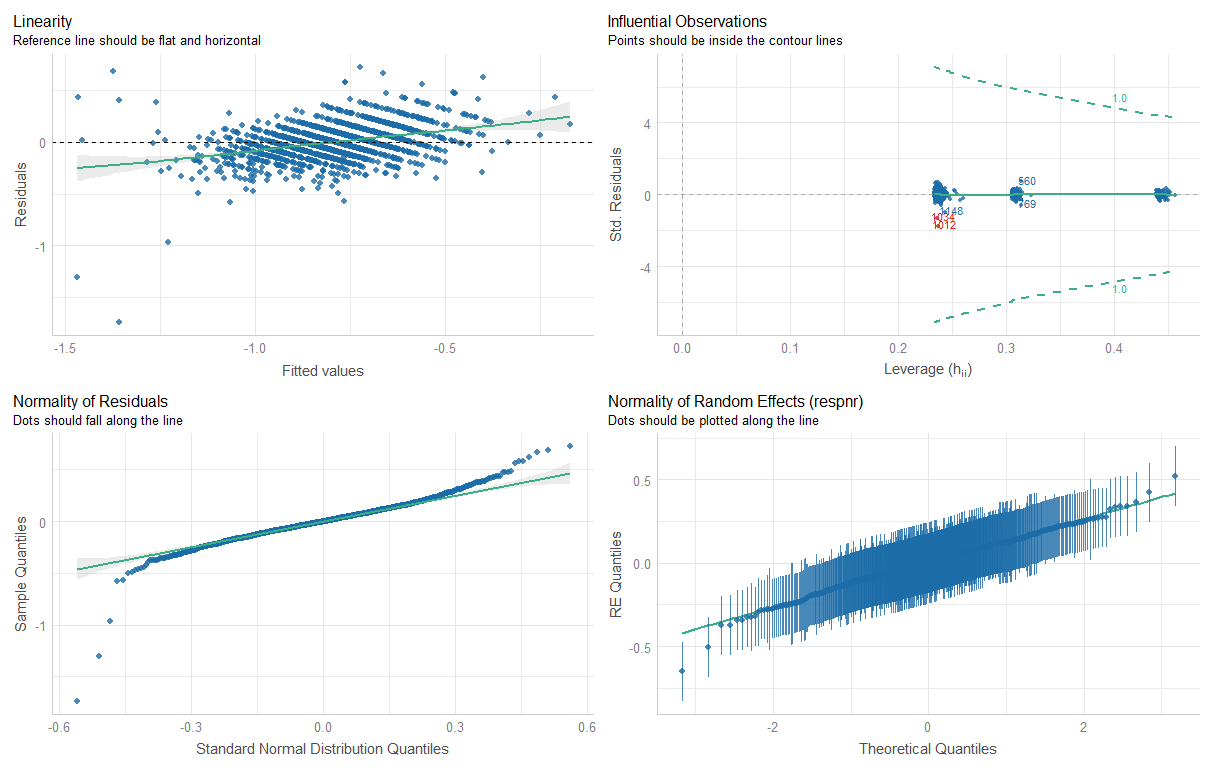


Figure S1D.
